# Nausea

**Published:** 1894-08

**Authors:** James B. Baird

**Affiliations:** Atlanta, Ga.


### ATLANTA
### MEDICAL AND SURGICAL JOURNAL.

Vol. XI. AUGUST, 1894. No. 6.

EDITED BY
LUTHER B. GRANDY, M.D., AND MILLER B. HUTCHINS, M.D.,
WITH THE CO-OPERATION OF
H. V. M. MILLER, M.D., LL.D., VIRGIL O. HARDON, M.D.,
FLOYD W. McRAE, M.D., ARTHUR G. HOBBS, M.D.,
AND H. R. SLACK, M.D., Ph.M.

### *ORIGINAL COMMUNICATIONS.*

### NAUSEA.*

By James B. Baird, M.D.,
Atlanta, Ga.

The term nausea, derived from a Greek word which signifies a ship, means sickness of the stomach with a tendency to vomit.

Nausea may be a symptom, more or less pronounced, of organic disease of the stomach, or of functional derangement of the stomach or of other portions of the digestive apparatus. It may be provoked by impressions received through the organs of special sense. Thus, sight, taste, smell, hearing and touch may stimulate emotions or thoughts, or may awaken associations which will at once develop a most distressing and, perchance, a protracted nausea, which may or may not be accompanied by vomiting.

Examples of nausea caused by riding in a railway train, by riding backward in a carriage, by swinging and by the rolling or the pitching motion of a sailing vessel or a steamship are familiar to

* Read before the Atlanta Obstetrical Society, May 10, 1894.

most persons. Mention of emetics administered by the mouth or hypodermatically, which cause nausea and vomiting by the impress which they exert upon the nervous system, should not be omitted.

Reflex influences of various kinds are potent factors in stirring up this depressing and dreaded disturbance. Intestinal, ovarian and testicular pain, menstrual disorders, violent injuries, etc., are the prime causes of much suffering from this source.

The sympathetic relations existing between the uterus and the stomach not infrequently cause nausea. This recognized relationship may cause nausea and vomiting to be the most prominent symptoms of pregnancy, and sometimes to constitute a most important, grave, or even fatal complication. Nausea alone, while it may prove extremely uncomfortable, is not dangerous unless nutrition should be seriously impaired in consequence of food being rendered obnoxious and habitually refused, or unless nutriment should be constantly rejected by the act of vomiting.

What may be termed the natural sympathy subsisting between the uterus and the stomach is exaggerated by the pregnant state, and this normal exaggeration, so to speak, may be enormously augmented and indefinitely intensified by uterine disorders, which thereby magnify, by many degrees, its reflective power. If this view is correct, it becomes very important, in severe cases, to employ judicious local treatment for the relief of the local disorder, which holds a causative relation to the excessive nausea.

I have had the opportunity of witnessing in numerous instances the most gratifying results of appropriate local treatment under these circumstances for the relief of inflammation, of erosion, or of ulceration of the os and cervix, and in cases, too, in which thoroughly respectable and capable practitioners had pronounced their sufferings a legitimate sequence of their condition, and counseled patience, only patience and fortitude, while the impending doom, if relief should be long delayed, might be read in the pallid and haggard face, and in the feeble and wasted form.

Even in threatening, occasionally in extreme, conditions from obstinate and long continued vomiting, with the consequent emaciation and prostration associated with a painful mental depression and anxiety bordering upon despair, prompt and decided relief often follows the application of the compound solution of iodine with or without the glycerine tampon and the hot vaginal douche, repeated, of course, as often and continued as long as may be required. Over and over again have I seen cases which appeared almost hopeless begin, within a few hours, to improve under this plan of treatment, and to so progress as to leave no room for doubt as to the cause of the amendment and of the ultimate recovery which speedily ensues.

I have referred to the compound solution of iodine as a favorite topical remedy in these cases. I prefer it to any other with which I am practically familiar. It is well borne and effective, and this is sufficient reason for commendation. I have not formed a favorable opinion of nitrate of silver as an application to the pregnant uterus. A solution of twenty grains to the ounce is sometimes energetically resented by the gravid womb, and I have seen severe uterine pains, with imminent danger of abortion, provoked by its use.

In all cases, then, of troublesome vomiting a thorough vaginal examination should be made, and, if the indications are found, appropriate local treatment should be employed. On the other hand, some of the most inveterate cases of vomiting present no appreciable uterine lesion. The organ is apparently perfectly healthy and engaged in the performance of its natural function, yet the life of the patient is placed in jeopardy by the constant rejection of everything that enters the stomach, and a fatal issue from rapid asthenia is imminent. Under these circumstances what is the duty of the medical adviser? To my mind the question admits of but one answer—abortion is the remedy. It will, with possibly few exceptions, yield prompt and complete relief. Failures, when they infrequently occur, are usually due, not to inefficiency, but to an unwise delay in the application of the remedy. Effective efforts for relief are withheld until such a degree of exhaustion is reached that recuperation is impossible. I have never had cause to regret the operation. I have had occasion, however, to lament its too long postponement or its entire rejection.

While I unreservedly approve and without hesitation adopt this mode of relief, and regard it as not only justifiable, but as absolutely obligatory in proper cases, I desire to emphasize the fact that I do not counsel reckless or inconsiderate resort to this valuable resource, and as a protection against censorious criticism, it is always important to have the concurrent assent of a consultant before undertaking this procedure. Other and simpler means, that do not involve the sacrifice of human life, as we must clearly understand that this procedure does, may suffice to secure relief. Patience and time, perchance only a short season of waiting, may show great improvement, followed by total cessation of the distressing phenomena. Certain well-known remedies and ordinary therapeutic resources undoubtedly bring relief in a large proportion of cases, or else they serve to bridge over the trying period until a change in the relation of the parts or the establishment of a nervous tolerance results in a partial or a total subsidence of the symptom. It is presumed, however, that this ground has been thoroughly covered and that the ordinary means of treatment have been exhausted. Radical measures have become imperative. The prime consideration is for the mother. The paramount question is : Shall we rescue the mother, rather than shall we sacrifice the fetus? The value of the two lives bears no comparison, and, in weighing the issue, it should be remembered that both are lost if the more important of the two is not saved.

The reflex influence above referred to may manifest itself in other directions besides, or instead of the production of excessive nausea and vomiting. I have seen intense paroxysms of pain in the abdominal walls and thighs, obstinate and uncontrollable except by large and often repeated doses of opium, promptly relieved by emptying the uterus. So have I seen immediate relief brought to a victim of the most desperate and alarming hysterical manifestations by the induction of abortion. All of these cases may be correlated. They are different in character, but have a common cause. The radical and specific remedy is the same.

Stretching the cervix, which has been recommended and indorsed by competent observers, has not been serviceable or successful in my hands. Either no good follows this manipulation, or abortion results from the interference with the uterus, and the resulting improvement must be attributed to that accident.

I consider the manner of producing abortion of some consequence. I prefer methods which simulate, as nearly as may be, the natural mode of discharging the uterine contents, and which inflict as little injury as possible upon the organ.

If interference is delayed until the woman reaches a point of imminent peril, no alternative remains but forcible dilatation and manual or instrumental extraction under chloroform; but if the inevitable is foreseen, if the danger signals are read aright, a gradual dilatation by means of the sound, of antiseptic tents aided and encouraged and made safe by the vaginal tampon, may stimulate expulsive pains, so that within a few hours, without haste, without anxiety and without violence, complete emptying may be secured. Occasionally, however, the tolerance to interference manifested by the uterus under these conditions is remarkable, and no reasonable amount of manipulation will provoke contractions. In that event, immediate dilatation and extraction becomes necessary, and whatever risk is incurred over and above that which pertains to the gentler and less alarming procedure, must be accepted. The most careful antiseptic precautions should invariably be taken.

